# Visual Attention and Hostile Media Perception in TikTok War News: An Eye-Tracking Study of Korean and Chinese Viewers

**DOI:** 10.3390/jemr19050120

**Published:** 2026-10-19

**Authors:** Mengqi Du, Ji-Eun Choi, Jawon Jang, Yifan Wang, Kyung Han You

**Affiliations:** 1 Department of Media and Communication Studies, Jeonbuk National University, 567 Baekje-daero, Deokjin-gu, Jeonju 54896, Republic of Korea; dumengqi08@gmail.com (M.D.); jihe5028@gmail.com (J.-E.C.); yf970616@gmail.com (Y.W.); 2 Institute for Data, Media and Communication Research (IDMCR), Jeonbuk National University, 567 Baekje-daero, Deokjin-gu, Jeonju 54896, Republic of Korea; 3 KoreaMedicare, 41 Mapo-daero 4da-gil, Mapo-gu, Seoul 04177, Republic of Korea

**Keywords:** hostile media perception, TikTok news, eye tracking, visual attention, cross-national news reception, short-form video

## Abstract

Short-form video platforms are major channels for conflict news, yet how viewers in different national contexts process such content and evaluate its sources remains unclear. This study combined eye tracking with pre- and post-exposure surveys among 124 university students (62 Korean and 62 Chinese) who viewed two native TikTok news posts about the Russia–Ukraine war, one from Korea’s MBN and one from China’s People’s Daily. Gaze was recorded at 60 Hz using a Tobii Pro Nano, with dynamic areas of interest defined for military imagery, people, and subtitles. Source-directed HMP, based on perceived ideological distance and source credibility, was assessed before and after exposure. Korean participants fixated longer on military imagery in both clips (d = 1.27 and 1.11), whereas Chinese participants attended longer to people in the Korean clip (d = −0.48). Subtitle-attention differences showed opposite directions across the two clips, with each group looking longer at subtitles in the clip produced in the other country. Baseline source-directed HMP was higher for the Korean outlet in both groups and was highest among Chinese participants. Across the fixed-order viewing session, HMP increased among Korean participants toward both outlets, whereas Chinese participants showed little change. Separately, among Korean participants, longer attention to people in the Chinese clip was associated with lower post-session HMP toward that outlet. These findings document cross-national differences in visual attention and associations between attention and source evaluation within two specific short-form conflict-news videos.

## 1. Introduction

In the digital era, short-form video platforms such as TikTok have reshaped how news is produced, distributed, and consumed. Native TikTok news posts typically combine vertical moving images, voice-over narration, on-screen text, and rapid editing within a brief audiovisual format. Recent evidence indicates that social and video platforms have become increasingly important channels for news use, particularly among younger audiences [1]. Research further suggests that young adults often encounter news through platform feeds rather than by directly visiting news organizations’ own websites or applications [2]. As platform-based news use has grown, news organizations have also expanded their presence on TikTok, adapting reporting practices to the platform’s visual and short-form conventions.

Compared with conventional television news, TikTok news posts present visual footage, subtitles, narration, and source cues within a compressed sequence. Viewers therefore process multiple message elements simultaneously while forming impressions of both the content and its source. Previous Korean research has examined hostile media perception in relation to domestic media and political entertainment [3]. Building on this work, the present study moves to a cross-national short-form news context by examining how Korean and Chinese university students attend to and evaluate native TikTok news posts produced by Korean and Chinese news outlets.

Building on this context, the present study examines how Korean and Chinese university students visually process native TikTok news posts and evaluate the news outlets that produced them. The study brings together three lines of work that have rarely been connected directly. Research on the visual hostile media phenomenon has shown that visual news content can be perceived as biased, but it has mainly relied on post-exposure evaluations rather than observing how viewers allocate attention during exposure [4]. Research on source and nationality cues has shown that outlet identity can shape perceived bias, but it has likewise relied mainly on self-report measures [5,6,7,8]. Eye-tracking studies in political and social media communication have examined attention to news content, but have rarely linked exposure-level gaze patterns to source-directed hostile media perception [9,10,11]. The present study connects these strands by combining eye tracking with pre- and post-session source evaluations in a cross-national comparison. Participants viewed two TikTok news posts about the Russia–Ukraine war, one produced by MBN in South Korea and the other by People’s Daily in China. The analysis focuses on attention to military imagery, people, and subtitles; changes in source-directed hostile media perception, understood as perceived media hostility toward one’s own position [12], across the viewing session; and associations between visual attention and outlet evaluation. By bringing these dimensions together, the study examines how visual attention and hostile media perception are related in the reception of short-form news from different national media contexts.

## 2. Literature Review

### 2.1. Short-Form Journalism and the Effects of Short-Form News Content

As video production and viewing became increasingly accessible, short-form video developed into a distinct content format [13]. TikTok has become a prominent platform for this form of audiovisual communication, while short-form video has also become increasingly integrated into broader social and video-platform environments. These developments have reshaped conventional patterns of video consumption and created new opportunities for news distribution.

Journalism has followed. Content-analytic work shows that news organizations have progressively adopted TikTok since 2019. In an analysis of verified news accounts worldwide, Vázquez-Herrero and colleagues found that news outlets used TikTok to inform audiences, position their brands, and adapt to platform-specific conventions in order to reach younger users [14]. As video-led platforms have become more central to online news use, established news organizations have increasingly needed to distribute content through formats that combine moving images, voice-over narration, on-screen text, and other platform-oriented features [1].

Beyond production, a growing body of reception research examines how audiences experience short-form news. Hendrickx found that young adults often encounter news through third-party platforms such as TikTok and Instagram rather than through news organizations’ own channels, and that they develop platform-specific expectations about how news should be presented [2]. Engagement is also associated with formal and affective characteristics of TikTok news videos. Cheng and Li found that negative sentiment and a greater use of second-person camera views were associated with higher levels of liking, commenting, and sharing, although these relationships differed between news publishers and other TikTok creators [15]. Recent global survey evidence likewise shows the growing importance of social and video networks for news use, particularly as online news video becomes increasingly concentrated on third-party platforms [1].

Together, this body of literature shows that TikTok news is shaped by platform-specific production practices and by audience engagement with audiovisual features. Existing research, however, has concentrated primarily on how news organizations adapt content to the platform and on audiences’ observable engagement with that content. Less is known about how viewers distribute visual attention across the multiple elements of a TikTok news post while viewing it, particularly when the news originates from different national media contexts. Eye tracking provides a means of examining this moment-to-moment allocation of attention to military imagery, people, and subtitles. Relating these exposure-level attention patterns to subsequent evaluations of the news outlets forms the basis for the present study.

### 2.2. War and Conflict Journalism in a Social Media Age

War reporting is among journalism’s most consequential domains because of its role in explaining a conflict’s origins, conduct, and aftermath and in offering audiences competing viewpoints. A long tradition in journalism studies shows that war coverage is rarely a neutral mirror: through framing, source selection, and emphasis, media shape how publics understand conflict and foreign policy [16]. In conflict settings, media narratives also become important sites through which political actors, journalists, and audiences negotiate competing interpretations of events.

Digital platforms have further transformed conflict reporting. Studies of social media coverage of the conflict in Eastern Ukraine show that visual framing can foreground particular actors, emotions, and interpretations, producing distinct and often nationally inflected representations of war [17]. The Russia–Ukraine war extended these dynamics to TikTok, where war-related videos became an important space for participation, testimony, advocacy, and commentary [18]. Research on young audiences further shows that TikTok can make distant conflict more familiar through the spectacularization of military technology, the incorporation of war into everyday platform content, and the aestheticization of conflict-related narratives [19].

These developments make the Russia–Ukraine war a particularly relevant context for examining the reception of TikTok news. Within a short audiovisual sequence, conflict-news posts combine moving images, subtitles, narration, human figures, and military-related footage, requiring viewers to distribute attention across several concurrent message elements. The meaning attached to these elements may also depend on the source of the report and the broader national context in which viewers encounter it. Examining the Korean and Chinese contexts therefore provides a basis for considering how the same international conflict is presented and received across different media environments.

### 2.3. The Russia–Ukraine War as a Transnational News Event: Korean and Chinese Contexts

The stimulus for this study is coverage of the Russia–Ukraine war, a conflict well-suited to examining identity-based perceptions of foreign news. Russia’s full-scale invasion began on 24 February 2022, escalating a confrontation whose roots lie in the 2014 annexation of Crimea and the war in the Donbas. It has since been extensively documented and contested on social media, where platforms such as TikTok became spaces for testimony, advocacy, and commentary on the war [18,19,20]. For the present design, the war provides a common international topic reported within distinct Korean and Chinese media contexts, allowing responses to news originating from the two countries to be compared within the same broader conflict.

Korea and China occupy markedly different positions toward the war, and their national media reflect this divergence. South Korea has aligned with the Western response: it condemned the invasion in United Nations votes, joined sanctions against Moscow, and provided humanitarian and non-lethal assistance to Ukraine—a stance maintained across both the progressive Moon and conservative Yoon administrations [21]. China, by contrast, has adopted what Xu and Zhao term “discursive strategic neutrality”: it refrained from outright condemnation of the invasion, attributed blame for escalating the conflict to the United States, NATO, and Europe, and sustained its strategic partnership with Moscow [22]. At the level of official discourse, Chinese foreign-ministry statements foregrounded mediation and multilateral cooperation while criticizing Western responses, echoing Russia’s narrative that Western actions had provoked the conflict [22].

These contrasting policy and media environments provide different source contexts through which the same international conflict may be encountered. Growing anti-China sentiment among the Korean public [23] may further shape expectations toward news outlets associated with the other national context. The Russia–Ukraine war therefore offers a suitable setting for examining whether Korean and Chinese participants differ in their visual attention to two short-form news videos and in their evaluations of the outlets that produced them.

### 2.4. Hostile Media Perception and Its Link to Conflict Coverage

The hostile media effect (HME) was, from its inception, a phenomenon of conflict coverage. In their foundational study of U.S. network reporting on the 1982 Beirut massacre, Vallone, Ross, and Lepper found that pro-Israeli and pro-Arab viewers each judged the same coverage as biased against their own side and the journalists as personally hostile [12]. Because the effect reflects a response to content rather than an objective property of the media, it is more precisely a hostile media perception [24]. Decades of replication have established its robustness: a meta-analysis of 34 studies reports a moderate effect across diverse contexts, with audience involvement as the principal moderator—perceived hostility intensifies as issue involvement rises [25]. Three mechanisms are commonly invoked—selective categorization, selective recall, and differential standards [12]—and the effect is generally understood as a function of partisanship and involvement [26] that also operates in a relative form, whereby opposing groups evaluate identical content differently [27].

Increasingly, HME is theorized as an intergroup phenomenon. Drawing on social identity theory, which holds that group membership organizes perception and judgment [28], research shows that the effect strengthens with ingroup identification: the more strongly individuals identify with their group, the more they read balanced coverage as hostile [29]. This identity-based account is especially apt for conflict reporting, where group boundaries are salient. Indeed, conflict coverage has been a recurrent testing ground for HMP. Beyond the Middle East cases [12,30], partisan perceptions of coverage of the Bosnian conflict were associated with social identification [31]; hostile media perceptions among Gaza settlers carried measurable consequences for trust in democratic institutions [32]; and perceptions of coverage of the China–Hong Kong conflict were shaped by cultural identity, not only by partisanship [33]. Together, these studies indicate that, in conflict settings, perceptions of media hostility can be shaped by social and national identities as well as by political affiliation.

Two developments extend this tradition in ways that are particularly relevant to short-form news. First, hostile media perception is not limited to verbal content. Research on the visual hostile media phenomenon shows that partisans can perceive even balanced sets of news images as biased against their own side, indicating that visual content is evaluated through viewers’ prior orientations [4]. This is especially relevant to image-led short-form video, in which visual and textual elements are presented simultaneously and change rapidly over time. Second, media evaluations are shaped not only by message content but also by cues surrounding its source. Research has shown that user comments can affect perceived bias and credibility in social media environments [34], while the identity and perceived affiliation of a news source can influence how audiences evaluate its reporting [5,6,7,8].

These developments converge in short-form news, where viewers encounter visual content, textual information, and recognizable source cues within the same compressed sequence. For this reason, evaluations of a news outlet may reflect expectations associated with the source as well as responses formed during exposure to a particular report. The present study therefore uses HMP in a source-directed sense: it examines how Korean and Chinese participants evaluated the Korean and Chinese news outlets before and after the viewing session, rather than asking them to judge whether either individual clip was biased against their own position. This distinction allows the study to connect visual attention during exposure with evaluations of identifiable news sources.

### 2.5. Nationality, Identity, and Cross-National Differences in News Reception

Nationality locates audiences within particular political, cultural, and media environments, but national membership and national identification are not identical constructs. Social identity theory and self-categorization theory propose that group membership becomes psychologically consequential when a particular collective identity is salient in a given context [35]. In international conflict coverage, cues relating to countries, institutions, and news sources may make national categories relevant to how audiences interpret a report. From this perspective, hostile media perception can emerge when a source is categorized as belonging to a relevant out-group, particularly when the associated group boundary is salient to the viewer [36].

Two additional processes may contribute to cross-national differences in news reception. Motivated reasoning—the tendency to process information so as to reach desired conclusions [37], such as those congruent with one’s group identity—has been applied in media research to explain how audience members may evaluate out-group sources as less credible and more hostile than in-group sources. The strength of identification also matters: experimental and survey research shows that perceptions of media bias can intensify with stronger ingroup identification, as demonstrated in coverage of inter-religious conflict [38] and of a contested social issue [29]. Reception may also be shaped by cultural proximity, as audiences often prefer content produced within familiar cultural environments over material from more distant national contexts [39].

Taken together, these perspectives suggest that cross-national differences in news reception may reflect a combination of source categorization, cultural proximity, motivated reasoning, and the varying salience of national identity. Accordingly, the present study uses participant nationality to compare audiences situated in different national contexts, while treating the strength of national identification as conceptually distinct from national membership. The analyses examine whether Korean and Chinese participants differ in their visual attention to short-form war news and in their evaluations of Korean and Chinese news outlets. They also consider whether the association between attention and source evaluation varies across the two participant groups.

### 2.6. Visual Attention and Eye Tracking

Because gaze position is tightly coupled to the locus of overt visual attention, recording eye movements is one of the most direct ways to observe how viewers sample a visual environment [40,41]. Eye movements comprise fixations, during which the eye is relatively stable and information is acquired (typically 100–500 ms), and saccades, the rapid relocations between fixations [40,41]. Visual attention is commonly divided into bottom-up processing, driven largely by stimulus features, and top-down, goal-directed processing shaped by task, memory, and context [42].

Video-based eye trackers estimate gaze position from the relationship between the pupil and the corneal reflection of infrared illumination [41]. Common measures include fixation count, fixation duration, and the spatial and temporal characteristics of scanpaths [43,44]. These measures indicate where and for how long viewers allocate overt visual attention during exposure. They do not, by themselves, establish how viewers understand or evaluate the attended material. Eye tracking therefore complements self-report measures by providing exposure-level information that viewers may be unable to report retrospectively [9,41].

In media research, areas of interest (AOIs) are used to assign fixations to predefined regions or categories of visual content. This approach makes it possible to compare attention to elements such as subtitles, human figures, and conflict-related imagery within a continuous audiovisual sequence. Related AOI-based approaches have been used to examine attention to social media content and political communication [9,10], while dynamic AOI methods have also been applied to videos of human faces [45]. In the present study, dynamic AOIs are used to examine how Korean and Chinese participants allocate attention to military-related imagery, people, and subtitles in two short-form news videos.

### 2.7. Research Questions

Bringing together the literature on hostile media perception, cross-national news reception, and visual attention, the present study examines three related dimensions of short-form conflict-news reception: the allocation of attention across visual elements, evaluations of Korean and Chinese news outlets across the viewing session, and the association between visual attention and source evaluation. Because prior evidence at the intersection of these areas remains limited, the study addresses the following research questions rather than proposing directional hypotheses.

**RQ1.**  *How do Korean and Chinese participants differ in their visual attention to military-related imagery, people, and subtitles across the Korean and Chinese news clips, and do these differences vary between the two clips?*

**RQ2.**  *How does source-directed hostile media perception toward the Korean and Chinese news outlets vary by participant nationality from pre-exposure to post-session measurement?*

**RQ3.**  *Does the association between attention to people in the Chinese-news clip and post-session source-directed hostile media perception toward the Chinese outlet differ between Korean and Chinese participants?*

## 3. Materials and Methods

### 3.1. Participants and Design

A total of 124 university students were recruited from a university in southwestern Korea in August 2026. The sample consisted of 62 Korean students and 62 Chinese international students. All participants provided written informed consent after being briefed on the eye-tracking procedure, and the protocol was approved by the Institutional Review Board of Jeonbuk National University (IRB No. JBNU 2026-07-037-001). Participants received a cultural gift voucher valued at KRW 5000 as compensation. No a priori power analysis was conducted; a sensitivity analysis indicates that with *n* = 62 per group and α = 0.05 (two-tailed), the design had 80% power to detect between-group differences of d ≥ 0.50.

The study employed a mixed design for the analysis of visual attention. Participant nationality (Korean vs. Chinese) served as the between-subjects grouping variable, and news clip (Korean vs. Chinese) served as the within-subject factor because each participant viewed both clips. All participants viewed the full duration of one Korean news clip and one Chinese news clip concerning the Russia–Ukraine war. Eye-movement measures were obtained separately for each clip. Hostile media perception toward each news outlet was assessed before exposure and again after participants had completed the two-clip viewing session.

### 3.2. Stimuli

To identify short-form news materials for the experiment, an initial pool of 50 highly viewed short-form videos was compiled from five Korean news outlets (KBS, SBS News, MBN, Yonhap News, and YTN) and three Chinese news outlets (People’s Daily, CCTV, and Xinhua News Agency). Videos concerning the Russia–Ukraine war were then identified because the conflict had received substantial coverage in both countries and provided a shared international topic for the cross-national comparison. Two videos were selected as the experimental stimuli: a 45.9-s clip produced by MBN in South Korea and a 21.6-s clip produced by People’s Daily in China.

Participants in both national groups viewed the same underlying Korean and Chinese video footage, but the subtitle language varied by participant group. Korean participants viewed Korean subtitles for both clips, whereas Chinese participants viewed Chinese subtitles for both clips. For the clip originating from the other national context, the subtitle text was translated into the participant’s native language while the original video footage was retained. Thus, participants could access the verbal information in their native language while viewing the same underlying visual material within each clip. The Korean- and Chinese-language subtitle versions were not equated for character count, line length, presentation rate, or screen coverage.

**Figure 1:**
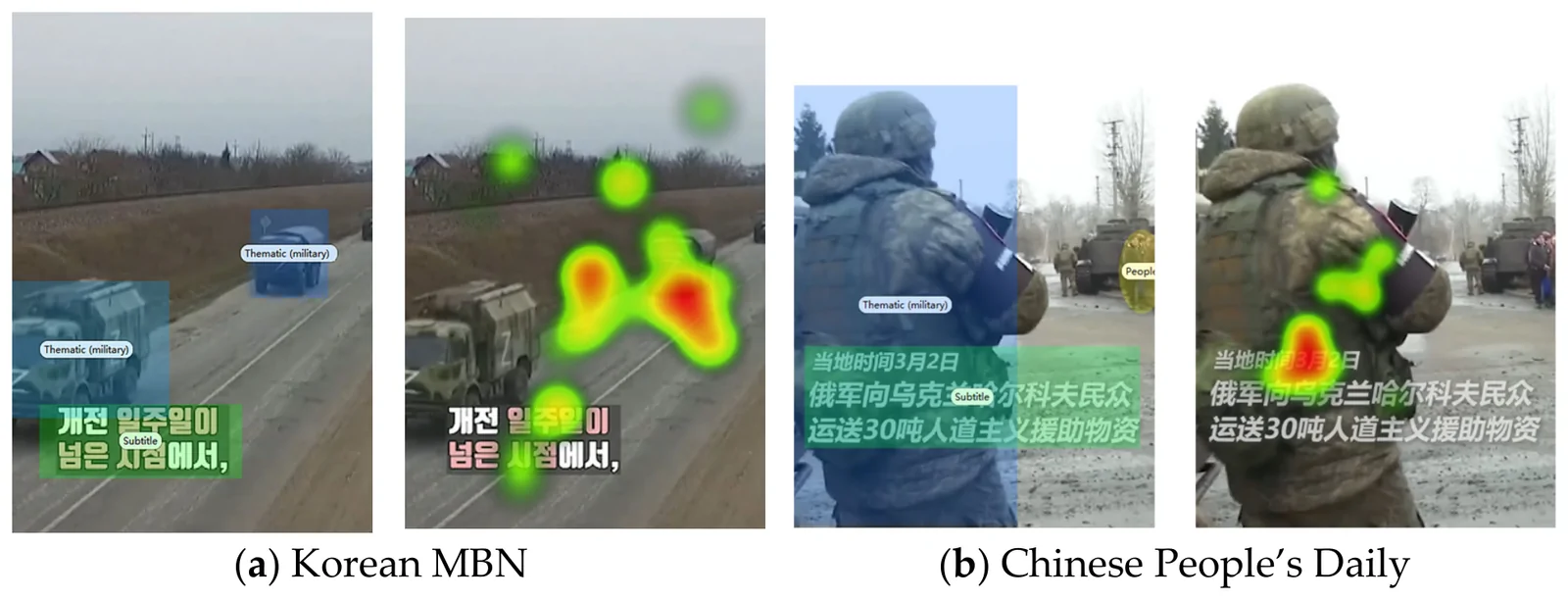
Representative frames from (**a**) the Korean MBN clip and (**b**) the Chinese People’s Daily clip. For each clip, the left frame shows the AOI boundary overlays present at that time point (military-related, people, and subtitle regions), and the right frame shows a group-level gaze heatmap at the corresponding time point. AOI boundaries were adjusted over time as visual elements moved or changed. Non-English text visible in the frames consists of the original on-screen subtitles of the stimulus clips, retained as presented to participants.

Because only one clip represented each outlet, source identity is confounded with the content, length, and production format of that clip. The two clips differed in length (45.9 s vs. 21.6 s) and in production format: the MBN clip relied mainly on moving footage with voice-over narration, whereas the People’s Daily clip was more image-led and carried much of its verbal information on screen. Intact native posts were used deliberately because source-directed evaluations refer to real outlets and are most meaningful when tied to content those outlets actually published; the cost is reduced control over content. Comparisons between participant groups within a clip hold the stimulus constant, whereas patterns observed across clips reflect both the source and the particular features of each video and are interpreted as descriptions of these two stimuli only (Figure 1).

### 3.3. Procedure

Data collection took place in August 2026 after Institutional Review Board approval was granted on 3 August 2026. Before the viewing session, participants were informed about the eye-tracking procedure and relevant precautions. They then completed a pre-exposure questionnaire covering their understanding of short-form news, country-related attitudes, and evaluations of the two news outlets.

Participants were seated approximately 65 cm from the display and completed a nine-point eye-tracker calibration. They then viewed both news clips in their entirety: the Korean MBN clip first, followed by the Chinese People’s Daily clip. This presentation order was the same for all participants. Gaze data were recorded throughout both clips. After the viewing session, participants completed a questionnaire that repeated the outlet-evaluation measures.

### 3.4. Apparatus and Eye-Tracking Data Processing

Gaze was recorded with a Tobii Pro Nano screen-based eye tracker mounted centrally beneath a 24-inch widescreen monitor and sampling binocularly at 60 Hz. Participants were seated in a quiet laboratory at a viewing distance of approximately 65 cm, so that the full screen subtended approximately 44.4° × 25.9° of visual angle and 1° corresponded to about 43 px. Because the stimuli were vertically oriented (9:16) platform videos, they were presented centered on the landscape display using the Fit to Screen setting at 608 × 1080 px, subtending approximately 14.7° × 25.9° and occupying 31.6% of the screen area; the surrounding letterbox region was rendered black and was excluded from all AOIs, so that the analyzed display area corresponds to the video frame rather than the full screen. Stimulus presentation and gaze recording were controlled by Tobii Pro Lab (version 1.217; Tobii AB, Danderyd, Sweden).

Before each recording, a standard nine-point calibration was performed in Tobii Pro Lab. Fixations were identified in Tobii Pro Lab using the Tobii I-VT (Fixation) gaze filter. A moving-median noise-reduction filter with a three-sample window was applied, and gaze velocity was calculated over a 20-ms window. The velocity threshold for fixation classification was 30°/s. Adjacent fixations separated by no more than 75 ms and 0.5° were merged, and fixations shorter than 60 ms were discarded. Gap-fill interpolation and the reclassification of discarded short fixations as saccades were disabled. The description of these processing settings was guided by the minimal reporting guideline for eye-tracking research [46].

Dynamic AOIs were defined in Tobii Pro Lab and re-drawn whenever the position or visibility of the relevant visual element changed across the course of each clip. Dynamic AOIs were drawn and adjusted frame by frame by two coders, with any discrepancies in category assignment or boundary placement resolved through discussion and consensus. Three AOI categories were coded: (1) thematic elements, comprising military-related content such as weapons, military vehicles, uniformed personnel, and damaged infrastructure; (2) people appearing in the video, treated as the background layer of the scene rather than as its thematic subject; and (3) on-screen subtitles. The thematic/background distinction matters for interpretation: the people AOI captures attention to the human surrounding of the report rather than to the conflict content that the report is nominally about. AOIs were allowed to overlap, and a fixation falling inside more than one AOI was credited to each applicable category; dwell times across the three categories are therefore not mutually exclusive and must not be summed as total viewing time.

**Figure 2:**
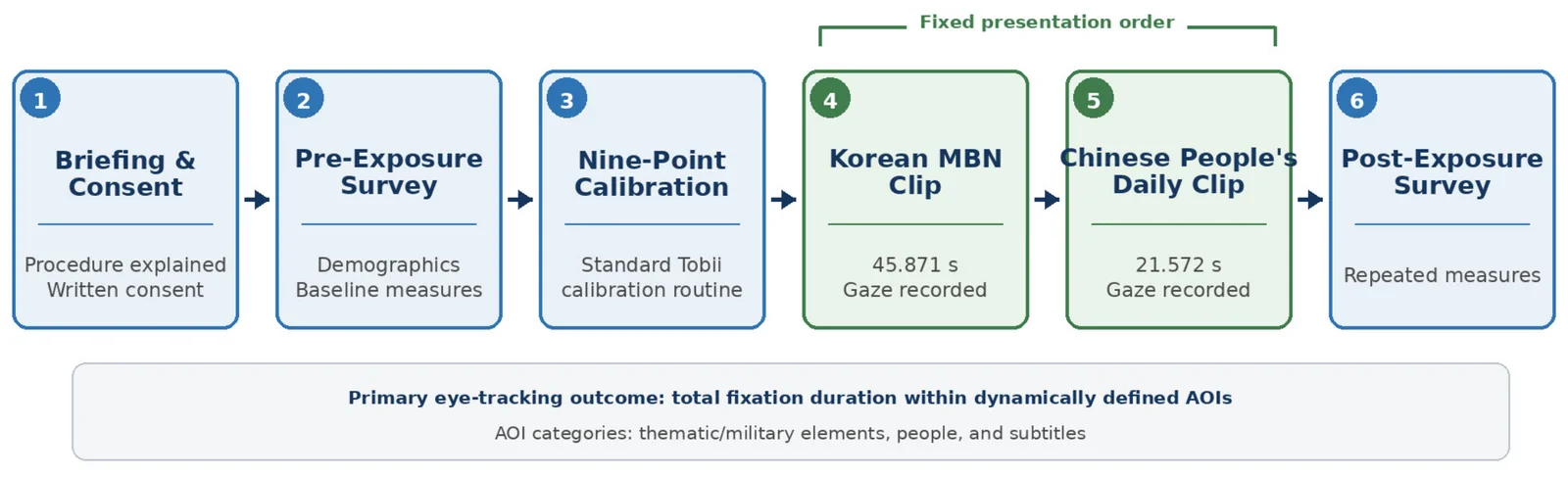
Procedure of the experiment.

Total fixation duration, in milliseconds, was computed for each AOI category and served as the primary attention measure. Data were processed in Tobii Pro Lab and analysed in IBM SPSS Statistics (Version 26). Figure 2 summarizes the sequence of the experimental session.

### 3.5. Measures

#### 3.5.1. Visual Attention (AOI)

Visual attention was operationalized as total fixation duration (in milliseconds) within each of the three dynamically defined AOI categories described in Section 3.4, computed separately for the Korean and the Chinese news clip.

#### 3.5.2. Hostile Media Perception (HMP)

Drawing on hostile media perception research [12] and the source-evaluation approach of Kim and Ha [3], we calculated a source-directed HMP index from perceived ideological distance and outlet credibility:

HMP = | participant’s ideology − perceived media ideology | × (5 − media credibility)

In both the pre-exposure and post-session surveys, participants reported their own political orientation and the perceived political orientation of each news outlet on 5-point scales ranging from 1 = very liberal to 5 = very conservative. They also rated the credibility of each outlet on a 5-point scale. The same HMP formula was applied at both time points. Credibility was reverse-scored as 5 − media credibility, ranging from 0 (highest credibility) to 4 (lowest credibility). The ideological-distance term ranges from 0 to 4; therefore, the index has a theoretical range of 0 to 16.

Higher index scores reflect a combination of greater perceived ideological distance from an outlet and lower perceived outlet credibility. As a source-directed measure, the index can capture evaluations formed before exposure as well as those reported after the viewing session. It differs from classic HMP measures that ask respondents to judge the bias of a particular report relative to their own position [12,27]. Because the index is multiplicative, a low score may arise from a small perceived ideological distance, high perceived credibility, or both.

## 4. Results

### 4.1. Participant Characteristics

The final sample consisted of 124 participants, 62 Korean students (50.0%) and 62 Chinese international students (50.0%), including 57 men (46.0%) and 67 women (54.0%). For the descriptive breakdown in Table 1, responses on the 5-point political-orientation scale were grouped as liberal or non-liberal, with the latter category combining moderate and conservative responses. The proportion classified as liberal was 51.6% among Korean participants and 40.3% among Chinese participants; this difference was not statistically significant, χ^2^(1) = 1.59, *p* = 0.207. The corresponding proportions were 29.8% among men and 59.7% among women, χ^2^(1) = 11.07, *p* < 0.001.

### 4.2. Visual Attention

Total fixation duration was compared between Korean and Chinese participants within each clip. Because the two groups differed in variance on several measures, all comparisons were conducted as Welch’s *t* tests, which do not assume homogeneity of variance; with equal group sizes the Welch statistic is numerically identical to the Student statistic, and only the degrees of freedom differ. Equality of variances was assessed with an F test on the two sample variances, and the ratios are reported in the note to Table 2. Given the modest sample size, 95% confidence intervals for the mean difference were obtained by bootstrapping with 5000 resamples, and effect sizes are reported as Cohen’s d computed with the pooled standard deviation. The six comparisons across three AOI categories and two clips were evaluated using the Holm–Bonferroni procedure. The *p* values reported in Table 2 are unadjusted; all comparisons reported as significant remained significant after correction.

For military-related AOIs, Korean participants showed significantly longer total fixation durations than Chinese participants in both the Korean-news clip (M = 18,300.84 ms, SD = 3419.50 vs. M = 14,332.68 ms, SD = 2786.79), t(117.2) = 7.08, *p* < 0.001, d = 1.27, 95% CI for the difference [2859, 5077] ms, and the Chinese-news clip (M = 4476.56 ms, SD = 1014.52 vs. M = 3403.40 ms, SD = 923.76), t(120.9) = 6.16, *p* < 0.001, d = 1.11, 95% CI [728, 1418] ms. For AOIs containing people, Chinese participants fixated significantly longer than Korean participants in the Korean-news clip (M = 17,588.60 ms, SD = 6934.24 vs. M = 14,705.84 ms, SD = 4918.65), t(110.0) = −2.67, *p* = 0.009, d = −0.48, 95% CI [−5020, −745] ms, whereas the groups did not differ significantly in the Chinese-news clip (M = 5341.68 ms, SD = 2090.61 vs. M = 4871.96 ms, SD = 1644.52), t(115.6) = −1.39, *p* = 0.167, d = −0.25.

For subtitle AOIs, the groups showed opposite patterns across the two clips. Chinese participants fixated subtitles longer in the Korean-news clip (M = 17,952.48 ms, SD = 8714.67 vs. M = 13,100.20 ms, SD = 5154.68), t(99.0) = −3.77, *p* < 0.001, d = −0.68, 95% CI [−7398, −2307] ms, whereas Korean participants fixated subtitles longer in the Chinese-news clip (M = 6864.76 ms, SD = 2424.32 vs. M = 4982.48 ms, SD = 3185.07), t(113.9) = 3.70, *p* < 0.001, d = 0.67, 95% CI [876, 2889] ms. Across the two clips, the observed between-group differences in subtitle fixation duration were in opposite directions: Chinese participants had longer fixation durations in the Korean-news clip, whereas Korean participants had longer fixation durations in the Chinese-news clip. All participants viewed subtitles in their native language. However, the Korean- and Chinese-language subtitle versions were not equated for character count, presentation rate, line length, or screen coverage, which may also have contributed to the observed differences. Because the two clips differed in length and in the on-screen time of each AOI, raw fixation durations are not on a common scale across clips, and no formal nationality × clip interaction was tested on them. The cross-clip subtitle pattern is therefore described in terms of the direction and size of the standardized within-clip effects.

### 4.3. Hostile Media Perception

Baseline HMP scores were summarized descriptively for the Korean and Chinese news outlets within each participant-nationality group (Table 3). Korean participants reported a higher mean baseline HMP score for the Korean outlet (M = 2.31, SD = 2.22) than for the Chinese outlet (M = 0.39, SD = 0.66). Chinese participants showed the same descriptive pattern (M = 3.62, SD = 2.20 for the Korean outlet; M = 0.58, SD = 0.93 for the Chinese outlet). The highest mean baseline HMP score was observed among Chinese participants evaluating the Korean outlet.

After viewing both clips in the fixed presentation order, participants completed the post-session HMP measures for each outlet. For each outlet, a difference score was calculated as post-session minus pre-exposure HMP; positive values indicate a higher score at the end of the viewing session. Because the post-session survey followed both clips, these scores describe change across the session.

As shown in Table 4, mean difference scores differed between the participant groups for both outlets. For the Korean news outlet, the mean difference was +1.03 (SD = 1.36) among Korean participants and −0.28 (SD = 1.01) among Chinese participants, t(112.5) = 6.08, *p* < 0.001, d = 1.09, 95% CI for the between-group difference [0.88, 1.74]. For the Chinese news outlet, the corresponding means were +1.44 (SD = 2.29) and −0.09 (SD = 1.33), respectively, t(98.2) = 4.55, *p* < 0.001, d = 0.82, 95% CI [0.86, 2.20]. Thus, Korean participants had positive mean difference scores for both outlets, whereas the Chinese participants’ mean difference scores were close to zero.

### 4.4. Attention to People × Nationality Interaction

Finally, a moderated regression examined whether the association between visual attention to people and post-session hostile media perception toward the Chinese news outlet differed by nationality. Because the visual elements were observed rather than manipulated, this model estimates an association, not an effect of attention on HMP. Gaze was recorded during exposure and HMP was measured afterwards, but this order of measurement does not establish the direction of the relationship. Consistent with RQ3, the model was specified to examine the association between attention and post-session HMP rather than change in HMP; pre-exposure HMP was therefore not entered as a covariate. Total fixation duration on people in the Chinese-news clip was entered in seconds, and nationality was coded 1 = Korean and 2 = Chinese. Nationality (b = −4.783, SE = 1.502, t = −3.18, *p* = 0.002), fixation duration on people (b = −1.379, SE = 0.435, t = −3.17, *p* = 0.002), and their interaction (b = 0.704, SE = 0.265, t = 2.66, *p* = 0.009) were significant (Table 5). Simple-slope analysis [47] indicated that longer fixation on people was associated with lower post-session HMP among Korean participants (b = −0.675 per second), whereas the association was essentially flat among Chinese participants (b = +0.029 per second).

**Figure 3:**
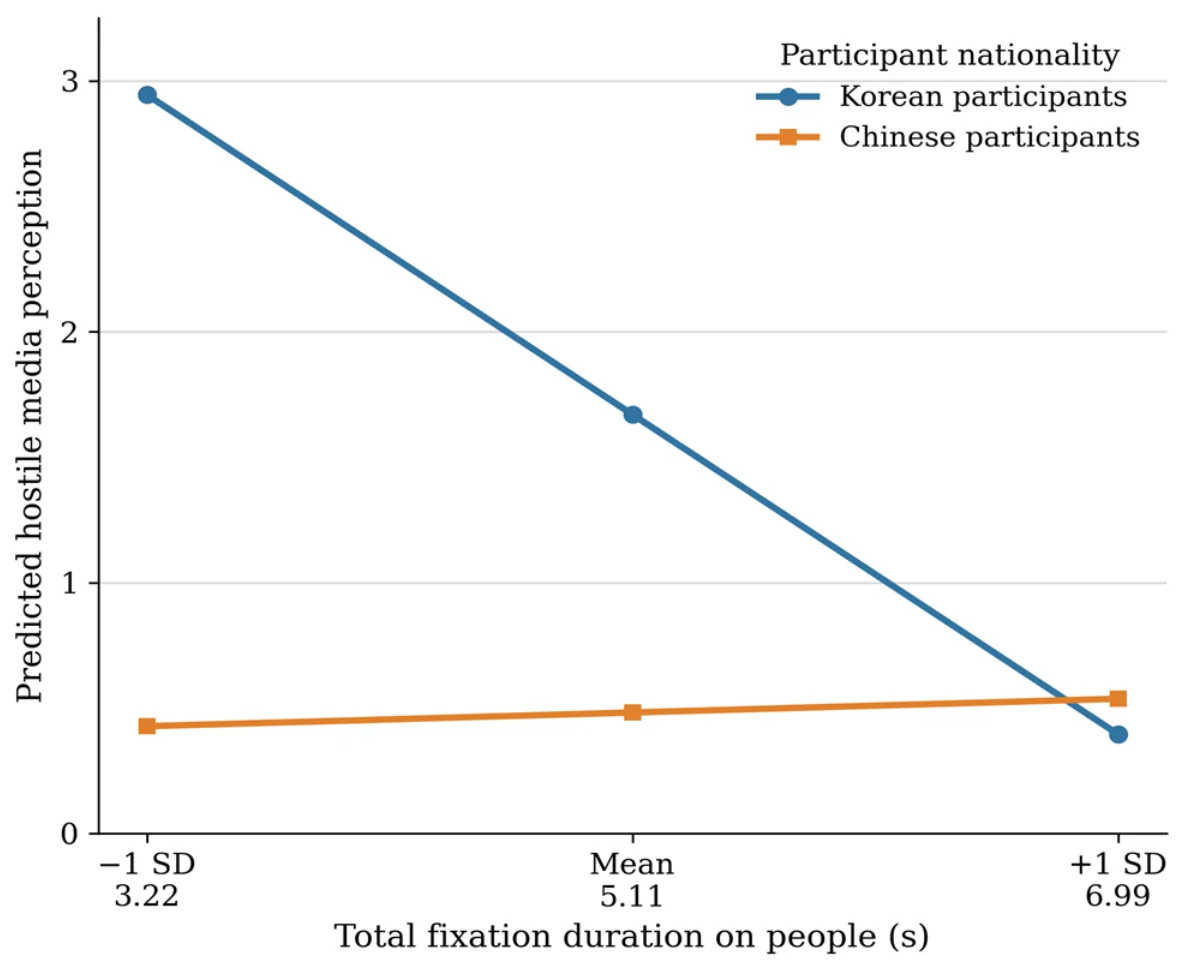
Predicted hostile media perception toward the Chinese news outlet as a function of total fixation duration on people, by participant nationality (from −1 SD to +1 SD).

Figure 3 plots the two simple slopes from one standard deviation below the mean fixation duration to one standard deviation above it. Using the overall mean (5.107 s) and standard deviation (1.888 s) of fixation duration in the people AOI of the Chinese-news clip, predicted HMP among Korean participants was 2.94 at −1 SD, 1.67 at the mean, and 0.40 at +1 SD. The corresponding predicted values among Chinese participants were 0.43, 0.48, and 0.54.

## 5. Discussion

The findings are best read at three levels of evidence. The first level is differences in visual attention between Korean and Chinese participants viewing the same clip. These are the most robust results because the stimulus was held constant within each comparison, although they remain specific to the two videos used in this study. The second level is the association between visual attention and source evaluation. This evidence rests on a moderated regression and should be interpreted as correlational and exploratory. The third level is a possible causal mechanism by which attention to particular visual elements may shape source evaluation. This mechanism was not directly tested. Accordingly, the interpretations below distinguish observed attention differences, attention–evaluation associations, and hypotheses for future experimental work.

### 5.1. Cross-National Patterns of Visual Attention in Short-Form War News

This study examined how Korean and Chinese university students visually processed and evaluated two short-form news videos concerning the Russia–Ukraine war. By integrating eye-tracking data with hostile media perception measures, the study captures both the allocation of visual attention during exposure and the evaluative judgments attached to news outlets. Eye tracking provides an exposure-level account of how viewers distribute attention across concurrent visual and verbal message layers, complementing retrospective self-report measures of media evaluation [10,11].

The most consistent attention pattern concerned military-related imagery. Korean participants recorded longer total fixation durations on military AOIs in both the Korean- and Chinese-news videos. This recurring pattern identifies military imagery as a central perceptual focal point for Korean participants across the two news contexts. Visual coverage of conflict can foreground military strength or civilian suffering to frame a war in contrasting ways [17]. The present findings suggest that Korean participants engaged more strongly with this conflict-centered visual layer while viewing short-form war news.

This pattern is relevant to the visual hostile media perspective, which emphasizes that media evaluations develop not only through verbal claims but also through visual cues, symbolic representations, and the social meanings attached to images [4]. In the present study, military imagery appears to have functioned as a prominent entry point through which Korean participants processed the geopolitical significance of the conflict. In video-based news, such cues also emerge, shift, and disappear across successive scenes, which the dynamic AOIs used here were designed to follow. The findings indicate that, for these two clips, Korean and Chinese participants differed in how they allocated gaze during exposure, in addition to differing in their self-reported evaluations of the outlets.

The subtitle findings reveal a complementary pattern. Chinese participants spent more time viewing subtitles in the Korean-news video, whereas Korean participants spent more time viewing subtitles in the Chinese-news video. Because subtitles were provided in the participants’ native language in every condition, this crossover cannot be reduced to a language barrier. Two features of the materials help account for it. First, the two clips distribute verbal information differently: the Korean clip carries its account primarily through voice-over narration over moving footage, while the Chinese clip is image-led and places most of the verbal content on screen, so the subtitle layer is doing more of the communicative work in the Chinese clip. Second, subtitle reading research shows that viewers’ gaze allocation to subtitles varies with subtitle presentation speed [48], and the density and timing of on-screen text may similarly affect how long viewers read it. One possible explanation is that viewers relied more on subtitles when the visual channel was less self-explanatory to them; however, differences between the two subtitle versions in length, timing, and presentation rate may also have contributed to this pattern.

Taken together, the attention findings suggest that, in these two clips, participants engaged with short-form war news through multiple perceptual routes. Military imagery became especially prominent for Korean participants across both clips, whereas subtitle attention shifted with the specific cross-national context of each video. These patterns suggest that, in the present stimuli, visual and verbal layers drew attention differently from the two participant groups.

### 5.2. Source Expectations and Outlet-Specific Hostile Media Perception

Before exposure, both participant groups had higher mean source-directed HMP scores for the Korean outlet than for the Chinese outlet. The highest mean was observed among Chinese participants evaluating the Korean outlet. These scores indicate that the two outlets were evaluated differently at baseline, although the product index does not show how perceived ideological distance and credibility contributed to each score.

Classic HMP research has focused largely on how audiences judge the bias of media content [12,27]. Research on source cues further shows that the identity and perceived affiliation of an outlet can inform such judgments [5,6,7,8], while evaluations may also vary with the context in which information is encountered [49]. In the present study, the baseline scores show that participants entered the viewing session with different evaluations of MBN and People’s Daily. These prior evaluations provide a context for interpreting their post-session responses.

Across the viewing session, Korean participants had positive mean HMP change scores for both outlets, whereas Chinese participants’ mean change scores were close to zero. The increase in the Korean group was thus observed for the domestic as well as the foreign outlet. Because all participants viewed the clips in the same order and completed the post-session measure only after both clips, the pattern cannot be attributed specifically to either video or to exposure to war coverage itself. Session progression, fatigue, habituation, and carryover remain possible explanations. These results therefore describe a session-level shift in source evaluation rather than the effect of either individual clip.

### 5.3. Human Figures as Socially Meaningful Visual Cues

The interaction between attention to people and HMP provides the most distinctive connection between visual processing and media evaluation. Chinese participants fixated people somewhat longer than Korean participants in the Korean-news clip (d = −0.48), while the two groups did not differ significantly in the Chinese-news clip. More consequential than these level differences is the association between attention and evaluation: in the Chinese-news clip, Korean participants who devoted more visual attention to people reported lower hostile media perception toward that outlet, whereas among Chinese participants this association was absent.

This finding moves beyond the simple question of whether viewers look more or less at people. Its theoretical significance lies in the evaluative role of human figures within conflict news. In the AOI scheme used here, people constitute the background layer of the scene rather than its thematic subject: the military imagery is what the report is nominally about, while the civilians, soldiers, bystanders and officials in frame form the human surround of that report. Such figures make geopolitical conflict concrete by connecting abstract political events to recognizable human agents. Research on dynamic face viewing shows that gaze toward people changes according to the informational and social relevance of facial and bodily cues within moving scenes [50]. In the present data, attention to people was associated with post-session source evaluation among Korean participants viewing the Chinese-news clip, although this model did not compare the people AOI directly with the thematic military AOI.

For Korean participants viewing the Chinese-news clip, longer attention to people was associated with weaker hostile media perception toward that outlet. One reading is that human figures functioned as a de-escalating cue: attending to the civilians, soldiers, and bystanders in the footage may have shifted processing away from the outlet’s national and institutional identity and toward the concrete human situation depicted, attenuating source-level suspicion. This would be consistent with accounts of individuation, in which depictions of specific persons reduce the salience of group boundaries. The interpretation remains post hoc, however: the design is correlational, HMP was measured only at the session level, and no process measure was collected.

Other explanations are equally compatible with the data. Participants who were already less hostile toward the outlet before viewing may have been more willing to attend to the people it depicted, which would reverse the direction of the association. Because pre-exposure HMP was not modeled, this possibility cannot be ruled out. Alternatively, a third factor, such as interest in the human consequences of the war, could underlie both longer attention to people and more favourable source evaluations. The difference in slopes between the two groups rules out explanations that would apply equally to both groups, but not explanations specific to one group. Distinguishing among these accounts requires manipulating the presence or prominence of human figures while holding the source constant.

The session-level change reported in Section 4.3 and the individual-level association reported in Section 4.4 concern different outcomes. Korean participants as a group showed higher HMP after the viewing session, whereas Table 5 indicates that, among Korean participants, greater attention to people in the Chinese-news clip was associated with lower post-session HMP toward the Chinese outlet.

This pattern speaks to the visual hostile media phenomenon, which proposes that audience orientations shape how the visual components of news content are perceived [4]. The present result extends that argument in an unexpected direction: longer attention to people was not accompanied by higher perceived hostility; instead, among Korean participants viewing the People’s Daily clip, it was associated with lower post-session HMP toward that outlet. It also complements research showing that hostile media judgments reflect the combined role of source cues, group identity, and message characteristics [7,8,38], adding a process-level dimension—source evaluation is connected not only to participants’ stated judgments but also to their visual engagement with socially salient elements during exposure.

The nationality × attention interaction indicates that the association between attention to human figures and source evaluation differed between the two participant groups for this clip. Whether human figures act as evaluative cues, and whether their role depends on national identification, remains to be tested experimentally. This offers a basis for future research on how visual social cues contribute to media-bias judgments in transnational news communication, and in particular for experimental designs that manipulate the presence and prominence of human figures rather than observing attention to them.

### 5.4. Theoretical and Practical Implications

#### 5.4.1. Theoretical Implications

This study contributes to hostile media perception research by linking source evaluation to visual processing in transnational short-form news. Previous HMP research has commonly treated perceived bias as a post-exposure evaluative outcome [12,27]. The present findings extend this approach by showing that cross-national differences emerged not only in HMP scores but also in attention to military imagery, subtitles, and human figures during exposure. This suggests that source evaluation in short-form news is connected to the perceptual organization of message cues as viewers process a visually complex environment [4], a process that eye-tracking methods make observable [10,11]. The direction of the attention–evaluation association was not uniform, however: greater attention to human figures was associated with lower, not higher, perceived hostility among Korean participants viewing the Chinese-news clip. This suggests that the visual hostile media account needs to specify which visual elements amplify and which attenuate group-based suspicion, rather than treating visual engagement as uniformly hostility-enhancing.

The findings also highlight the importance of source-specific visual processing. The Korean and Chinese outlets occupied different evaluative positions before exposure, indicating that source identity and associated national or ideological expectations shape how audiences approach transnational news [5,6,7,8]. In addition, the association between attention to people and HMP in the Chinese-news stimulus suggests that human figures can function as socially meaningful cues through which viewers interpret the orientation of a news source. Together, these results offer an exposure-level, correlational complement to post-exposure accounts of HMP by identifying visual elements whose role in source evaluation merits experimental testing.

#### 5.4.2. Practical Implications

The attention patterns observed in the two clips offer a practical reason to examine how short-form news presents information to audiences from different national contexts. Korean and Chinese participants did not allocate gaze uniformly to military imagery, people, and subtitles. For news producers, audience testing may help establish whether viewers notice the visual and textual information that a report is intended to convey.

Subtitles merit particular attention when a video is adapted for viewers using different languages. Presentation speed affects how viewers read subtitles [48], and subtitle length, timing, and screen position may likewise affect how readily viewers can follow the report. Comparing the readability and screen coverage of different subtitle versions would help producers assess how translation choices alter the viewing experience.

### 5.5. Limitations and Directions for Future Research

Several limitations should be considered when interpreting the findings. First, the study used two intact short-form news videos, with only one clip representing each outlet. Although this retained the videos’ original footage and editorial features, source identity could not be separated from the specific content, duration, narration, and visual composition of each clip. The findings therefore concern these two stimuli and should not be generalized to Korean and Chinese news more broadly. Future studies could use multiple matched clips from each source and vary source labels separately from video content.

Second, presentation order was fixed for all participants: the Korean MBN clip was always shown before the Chinese People’s Daily clip. Clip identity was thus confounded with its position in the viewing sequence. Fatigue, habituation to the eye-tracking task, and carryover from the first clip cannot be ruled out as influences on responses to the second. Because the post-session HMP measure followed both clips, changes across the session also cannot be attributed to either clip individually. Future studies should counterbalance clip order and consider measuring source evaluations after each clip.

Third, the sample consisted of Korean students and Chinese international students recruited from one university in South Korea. The results may not extend to broader audiences in either country. In addition, nationality was used to compare participant groups, but the strength of national identification was not measured. Because individuals of the same nationality can identify with their national group to varying degrees, the identity-related processes discussed in the theoretical framework remain untested, and the group differences reported here may reflect differences in media environments, cultural familiarity, issue involvement, or prior attitudes, as well as differences in identity. Future research could recruit participants in both countries and assess national identification, source familiarity, issue involvement, prior conflict attitudes, and broader media trust.

Fourth, the visual elements were observed within the videos rather than experimentally manipulated. An association between attention to an AOI and HMP cannot establish that attending to that element changed source evaluation. AOI boundaries also changed with the footage, could overlap, and differed in area across categories; raw fixation durations should therefore not be compared directly between military imagery, people, and subtitles. Furthermore, the two language versions of the subtitles were not matched for character count, presentation rate, line length, or screen coverage. These differences may have contributed to the subtitle-attention patterns. Future studies could use more closely matched subtitles and experimentally vary selected visual elements.

Fifth, the moderated regression reported in Table 5 examined post-session HMP toward the Chinese outlet and therefore does not show whether attention was related to change in HMP. Future studies could model change directly, either by including baseline HMP as a covariate or by using pre–post difference scores as the dependent variable, preferably with multi-item HMP measures whose reliability can be assessed.

Sixth, the HMP index measured evaluations of news sources through the product of perceived ideological distance and reverse-coded outlet credibility. It does not measure perceived bias in a particular report and is therefore not directly comparable with classic content-directed HMP measures. A low score can result from a small ideological distance, high credibility, or both; the product does not distinguish between these explanations. This ambiguity is especially relevant to the low baseline means for the Chinese outlet. As the index is not a conventional multi-item scale, internal-consistency reliability is not applicable. Future studies should examine its validity, report both components separately, and include a validated content-directed HMP measure.

Finally, data were collected in August 2026 using archived clips concerning an earlier period of the Russia–Ukraine war. Responses to these materials should not be taken as a description of the current short-form news environment.

## 6. Conclusions

This study examined visual attention and hostile media perception among Korean and Chinese university students exposed to Korean and Chinese short-form news videos about the Russia–Ukraine war. Eye-tracking results showed that Korean participants devoted greater attention to military-related imagery across both clips, that Chinese participants attended longer to people in the Korean clip, and that subtitle-attention differences showed opposite directions across the two clips. Baseline HMP scores were higher for the Korean outlet in both participant groups, with the highest scores among Chinese participants. After viewing, Korean participants reported higher HMP scores for both outlets, whereas Chinese participants’ evaluations remained comparatively stable.

The findings indicate that hostile media perception in short-form news is associated with both source-related expectations and attention to dynamic message cues. Across the fixed-order viewing session, Korean participants’ source-directed HMP scores increased for both the domestic and the foreign outlet; because presentation order was fixed and HMP was measured only after both clips, this change cannot be attributed to either clip or to exposure itself. Separately, among Korean participants viewing the Chinese-news clip, greater attention to people was associated with lower post-session HMP toward the Chinese outlet, suggesting that human figures may play a role in source evaluation, a possibility that requires experimental confirmation. By combining eye tracking with source-directed evaluation measures, this study offers an exposure-level perspective on how these viewers attended to visual layers of short-form war news and evaluated the outlets that produced it.

These findings come from Korean and Chinese university students recruited at one Korean university, two short-form clips, and a single geopolitical topic. They should therefore be read as evidence about how these viewers attended to and evaluated these two clips, not as a general account of how Korean and Chinese audiences receive news. What may carry over to other topics and media systems is the approach: pairing attention to specific visual layers during exposure with source-directed evaluations measured before and after viewing. Its value will depend on replication with multiple stimuli, more varied samples, and other contested issues.

## Figures and Tables

**Table 1:** Political orientation by nationality and gender.

Characteristic	Category	Liberal, *n* (%)	Non-Liberal, *n* (%)	Total, *n* (%)
Nationality	Korean	32 (51.6)	30 (48.4)	62 (100.0)
	Chinese	25 (40.3)	37 (59.7)	62 (100.0)
Gender	Male	17 (29.8)	40 (70.2)	57 (100.0)
	Female	40 (59.7)	27 (40.3)	67 (100.0)
Overall		57 (46.0)	67 (54.0)	124 (100.0)

Note. Percentages for the nationality and gender rows are row percentages; overall percentages are based on the full sample (N = 124). Political orientation was grouped as liberal versus non-liberal for the descriptive comparisons in this table. The HMP index was calculated using the original 5-point political-orientation responses rather than these two categories. Ideology did not differ by nationality, χ^2^(1) = 1.59, *p* = 0.207, but did differ by gender, χ^2^(1) = 11.07, *p* < 0.001.

**Table 2:** Total fixation duration (ms) within dynamically defined AOIs, by participant nationality and clip.

AOI	Stimulus	Group	M (ms)	SD	Welch’s t	df	*p*	d	95% CI
Thematic (military)	Korean news	Korean	18,300.84	3419.50	7.08	117.2	<0.001	1.27	[2859, 5077]
		Chinese	14,332.68	2786.79					
	Chinese news	Korean	4476.56	1014.52	6.16	120.9	<0.001	1.11	[728, 1418]
		Chinese	3403.40	923.76					
People	Korean news	Korean	14,705.84	4918.65	−2.67	110.0	0.009	−0.48	[−5020, −745]
		Chinese	17,588.60	6934.24					
	Chinese news	Korean	4871.96	1644.52	−1.39	115.6	0.167	−0.25	[−1138, 199]
		Chinese	5341.68	2090.61					
Subtitle	Korean news	Korean	13,100.20	5154.68	−3.77	99.0	<0.001	−0.68	[−7398, −2307]
		Chinese	17,952.48	8714.67					
	Chinese news	Korean	6864.76	2424.32	3.70	113.9	<0.001	0.67	[876, 2889]
		Chinese	4982.48	3185.07					

Note. *n* = 62 per group. Welch’s *t* tests are reported because group variances were unequal for several comparisons; with equal group sizes the Welch statistic equals the Student statistic and only the degrees of freedom differ. Variance ratios: military/Korean clip F(61,61) = 1.51, *p* = 0.113; military/Chinese clip F = 1.21, *p* = 0.466; people/Korean clip F = 1.99, *p* = 0.008; people/Chinese clip F = 1.62, *p* = 0.063; subtitle/Korean clip F = 2.86, *p* < 0.001; subtitle/Chinese clip F = 1.73, *p* = 0.035. Effect sizes are Cohen’s d computed with the pooled SD; negative values indicate longer dwell time among Chinese participants. Confidence intervals are for the between-group mean difference, from 5000 bootstrap resamples. All *p* values below 0.05 survive Holm–Bonferroni correction across the six comparisons.

**Table 3:** Baseline hostile media perception scores for the Korean and Chinese news outlets, by participant nationality.

Participant Group	Outlet	M	SD
Korean	Korean news	2.31	2.22
	Chinese news	0.39	0.66
Chinese	Korean news	3.62	2.20
	Chinese news	0.58	0.93

Note. *n* = 62 per group. Values are descriptive statistics.

**Table 4:** Change in hostile media perception from pre-exposure to post-session measurement (post − pre), by participant nationality.

Outlet	Participant Group	M	SD	Welch’s t	df	*p*	d	95% CI
Korean news	Korean	1.03	1.36	6.08	112.5	<0.001	1.09	[0.88, 1.74]
	Chinese	−0.28	1.01					
Chinese news	Korean	1.44	2.29	4.55	98.2	<0.001	0.82	[0.86, 2.20]
	Chinese	−0.09	1.33					

Note. *n* = 62 per group. Difference scores were calculated as post-session HMP minus pre-exposure HMP; positive values indicate an increase in perceived source hostility across the session. For each outlet, the *t*-test compares difference scores between Korean and Chinese participants; Welch’s test is reported because group variances were unequal (Korean outlet F(61,61) = 1.82, *p* = 0.021; Chinese outlet F = 2.94, *p* < 0.001).

**Table 5:** OLS moderated regression predicting post-session hostile media perception toward the Chinese news outlet from attention to people, nationality, and their interaction.

Predictor	Estimate	SE	t	*p*
Intercept (c)	9.90	2.29	4.33 ***	<0.001
Nationality (a)	−4.78	1.50	−3.18 **	0.002
Total fixation duration for people (b)	−1.38	0.44	−3.17 **	0.002
Interaction term (a × b)	0.70	0.27	2.66 **	0.009
F(3, 120)	18.90 ***			
R^2^	0.32			

Note. N = 124. The dependent variable is post-session hostile media perception toward the Chinese news outlet. Fixation duration on people is total fixation duration within the people AOI of the Chinese-news clip, in seconds. Coefficients were estimated using ordinary least squares (OLS). Results are interpreted as exploratory associations with post-session HMP (see Section 4.4). Simple slopes: b = −0.675 per second among Korean participants and b = +0.029 per second among Chinese participants. ** *p* < 0.01, *** *p* < 0.001.

## Data Availability

The data supporting the findings of this study, including de-identified survey data, AOI definitions, and aggregated AOI-level fixation-duration data, are available from the corresponding author upon reasonable request. Raw participant-level gaze data are not publicly available due to participant privacy and ethical restrictions.

## References

[1] Egan J., Robertson C.T., Ross Arguedas A., Newman N., Nielsen R.K., Mukherjee M., Fletcher R. (2026). Reuters Institute Digital News Report 2026.

[2] Hendrickx J. (2025). “Normal news is boring”: How young adults encounter and experience news on Instagram and TikTok. New Media Soc..

[3] Kim Y.-J., Ha S.-T. (2018). Perception of media bias: Comparison of HMP for TV news and political entertainment. Locality Commun..

[4] Matthes J., Schmuck D., von Sikorski C. (2023). In the eye of the beholder: A case for the visual hostile media phenomenon. Commun. Res..

[5] Arpan L.M., Raney A.A. (2003). An experimental investigation of news source and the hostile media effect. J. Mass Commun. Q..

[6] Gunther A.C., Liebhart J.L. (2006). Broad reach or biased source? Decomposing the hostile media effect. J. Commun..

[7] Golan G.J., Waddell T.F., Barnidge M. (2021). Competing identity cues in the hostile media phenomenon: Source, nationalism, and perceived bias in news coverage of foreign affairs. Mass Commun. Soc..

[8] Gunther A.C., McLaughlin B., Gotlieb M.R., Wise D. (2017). Who says what to whom: Content versus source in the hostile media effect. Int. J. Public Opin. Res..

[9] Vraga E., Bode L., Troller-Renfree S. (2016). Beyond self-reports: Using eye tracking to measure topic and style differences in attention to social media content. Commun. Methods Meas..

[10] Kruikemeier S., Lecheler S., Boyer M.M. (2018). Learning from news on different media platforms: An eye-tracking experiment. Polit. Commun..

[11] King A.J., Bol N., Cummins R.G., John K.K. (2019). Improving visual behavior research in communication science: An overview, review, and reporting recommendations for using eye-tracking methods. Commun. Methods Meas..

[12] Vallone R.P., Ross L., Lepper M.R. (1985). The hostile media phenomenon: Biased perception and perceptions of media bias in coverage of the Beirut massacre. J. Pers. Soc. Psychol..

[13] Lee J. (2020). A study on types of short-form video contents. Humanit. Contents.

[14] Vázquez-Herrero J., Negreira-Rey M.-C., López-García X. (2022). Let’s dance the news! How the news media are adapting to the logic of TikTok. Journalism.

[15] Cheng Z., Li Y. (2024). Like, comment, and share on TikTok: Exploring the effect of sentiment and second-person view on the user engagement with TikTok news videos. Soc. Sci. Comput. Rev..

[16] Entman R.M. (2004). Projections of Power: Framing News, Public Opinion, and U.S. Foreign Policy.

[17] Makhortykh M., Sydorova M. (2017). Social media and visual framing of the conflict in Eastern Ukraine. Media War Confl..

[18] Kalnes Ø., Bjørge N.M. (2025). ‘So, we have occupied TikTok’: Ukrainian women in #ParticipativeWar. Media War Confl..

[19] Colombo E., Giorgi G., Rebughini P.A. (2025). When the war strikes back: TikTok’s domestication of the Russo-Ukrainian conflict among youth. Media Cult. Soc..

[20] BBC News Korea (2022). Ukraine Crisis: ‘I Want Everyone to Know About This’—A Ukrainian Woman Sharing the War on TikTok. https://www.bbc.com/korean/international-60636081.

[21] Pacheco Pardo R., Kim S. (2023). South Korea: Siding with the West and distancing from Russia. Int. Polit..

[22] Xu Y., Zhao X. (2025). China’s discursive strategic neutrality position in the Russo-Ukrainian war: Insights from China’s strategic narratives. J. Contemp. China.

[23] Kang S.-J. (2022). The spread of anti-China sentiment?: An analysis of changes in South Koreans’ feelings and perceptions toward China. J. Mod. China Stud..

[24] Perloff R.M. (2015). A three-decade retrospective on the hostile media effect. Mass Commun. Soc..

[25] Hansen G.J., Kim H. (2011). Is the media biased against me? A meta-analysis of the hostile media effect research. Commun. Res. Rep..

[26] Gunther A.C. (1992). Biased press or biased public? Attitudes toward media coverage of social groups. Public Opin. Q..

[27] Gunther A.C., Schmitt K. (2004). Mapping boundaries of the hostile media effect. J. Commun..

[28] Tajfel H., Turner J.C., Austin W.G., Worchel S. (1979). An integrative theory of intergroup conflict. The Social Psychology of Intergroup Relations.

[29] Hartmann T., Tanis M. (2013). Examining the hostile media effect as an intergroup phenomenon: The role of ingroup identification and status. J. Commun..

[30] Perloff R.M. (1989). Ego-involvement and the third person effect of televised news coverage. Commun. Res..

[31] Matheson K., Dursun S. (2001). Social identity precursors to the hostile media phenomenon: Partisan perceptions of coverage of the Bosnian conflict. Group Process. Intergroup Relat..

[32] Tsfati Y., Cohen J. (2005). Democratic consequences of hostile media perceptions: The case of Gaza settlers. Harv. Int. J. Press..

[33] Cao B., Chen Z., Huang Y., Lo W.H. (2014). Conflict between Mainland Chinese and Hong Kongers: A social identity perspective in explaining the hostile media phenomenon and the third-person effect. J. Appl. J. Media Stud..

[34] Gearhart S., Moe A., Zhang B. (2020). Hostile media bias on social media: Testing the effect of user comments on perceptions of news bias and credibility. Hum. Behav. Emerg. Technol..

[35] Turner J.C., Hogg M.A., Oakes P.J., Reicher S.D., Wetherell M.S. (1987). Rediscovering the Social Group: A Self-Categorization Theory.

[36] Reid S.A. (2012). A self-categorization explanation for the hostile media effect. J. Commun..

[37] Kunda Z. (1990). The case for motivated reasoning. Psychol. Bull..

[38] Ariyanto A., Hornsey M.J., Gallois C. (2007). Group allegiances and perceptions of media bias: Taking into account both the perceiver and the source. Group Process. Intergroup Relat..

[39] Straubhaar J.D. (1991). Beyond media imperialism: Asymmetrical interdependence and cultural proximity. Crit. Stud. Mass Commun..

[40] Rayner K. (2009). The 35th Sir Frederick Bartlett lecture: Eye movements and attention in reading, scene perception, and visual search. Q. J. Exp. Psychol..

[41] Holmqvist K., Nyström M., Andersson R., Dewhurst R., Jarodzka H., van de Weijer J. (2011). Eye Tracking: A Comprehensive Guide to Methods and Measures.

[42] Corbetta M., Shulman G.L. (2002). Control of goal-directed and stimulus-driven attention in the brain. Nat. Rev. Neurosci..

[43] Goldberg J.H., Kotval X.P. (1999). Computer interface evaluation using eye movements: Methods and constructs. Int. J. Ind. Ergon..

[44] Blascheck T., Kurzhals K., Raschke M., Burch M., Weiskopf D., Ertl T. (2017). Visualization of eye tracking data: A taxonomy and survey. Comput. Graph. Forum.

[45] Hessels R.S., Benjamins J.S., Cornelissen T.H.W., Hooge I.T.C. (2018). A validation of automatically-generated areas-of-interest in videos of a face for eye-tracking research. Front. Psychol..

[46] Dunn M.J., Alexander R.G., Amiebenomo O.M., Arblaster G., Atan D., Erichsen J.T., Ettinger U., Giardini M.E., Gilchrist I.D., Hamilton R. (2024). Minimal reporting guideline for research involving eye tracking (2023 edition). Behav. Res. Methods.

[47] Hayes A.F. (2022). Introduction to Mediation, Moderation, and Conditional Process Analysis: A Regression-Based Approach.

[48] Szarkowska A., Gerber-Morón O. (2018). Viewers can keep up with fast subtitles: Evidence from eye movements. PLoS ONE.

[49] Gunther A.C., Miller N., Liebhart J.L. (2009). Assimilation and contrast in a test of the hostile media effect. Commun. Res..

[50] Võ M.L.-H., Smith T.J., Mital P.K., Henderson J.M. (2012). Do the eyes really have it? Dynamic allocation of attention when viewing moving faces. J. Vis..

